# “Please don’t expose me”: students’ perceptions of psychological safety during peer feedback on presentations

**DOI:** 10.3389/fpsyg.2026.1743621

**Published:** 2026-04-02

**Authors:** Suzanne Mohamed Arafa, Sayed Ibrahim Ali

**Affiliations:** 1 Mohammed Jaber Al Ansari College for Teachers, University of Bahrain, Manama, Bahrain; 2 Educational Psychology Department, Faculty of Education, Capital University (Helwan) , Cairo, Egypt

**Keywords:** oral presentations, peer feedback, psychological safety, qualitative phenomenology, Saudi Arabia, student perceptions

## Abstract

**Introduction:**

Psychological safety is essential for meaningful participation in peer learning activities, particularly those involving public performance and evaluation. While peer feedback on oral presentations is widely used in health professions education to enhance evaluative judgment and communication skills, limited research has examined how students experience psychological safety during such feedback encounters. In culturally relational and gender-sensitive contexts, public critique may be interpreted as social exposure rather than academic support. This study explored how undergraduate health students perceive and negotiate psychological safety when giving and receiving peer feedback on oral presentations.

**Methods:**

An interpretive (hermeneutic) phenomenological design was adopted. Eighteen undergraduate health and health-related students at King Faisal University participated in semi-structured interviews. Participants had recently delivered oral or case-based presentations followed by peer feedback. Interviews were audio-recorded, transcribed verbatim, and analyzed using reflexive thematic analysis following Braun and Clarke’s six-phase framework. Rigor was supported through reflexive journaling, peer debriefing, member checking, and maintenance of an audit trail.

**Results:**

Four interconnected themes were generated: (1) Public feedback as social threat, where public critique amplified vulnerability and concerns about loss of face; (2) Instructor as safety broker, highlighting the teacher’s role in framing, moderating, and shielding students from harsh commentary; (3) Relational calculus in giving feedback, where comments were filtered through friendship, hierarchy, and gender sensitivities; and (4) Making peer feedback safe and useful, emphasizing the value of anonymous or written channels and growth-oriented framing. Students did not reject peer feedback itself but resisted formats that heightened social risk.

**Discussion:**

Peer feedback on presentations is experienced as a socially negotiated event rather than a purely pedagogical activity. Psychological safety determines whether feedback is received as developmental or exposing. Structured moderation, culturally sensitive facilitation, and protected feedback channels enable rigorous yet dignity-preserving peer learning. Designing psychologically safe feedback practices is essential for preparing reflective and communicative health professionals.

## 1 Background

Psychological safety has emerged as a key construct in understanding how learners participate, disclose difficulties, and take interpersonal risks in educational settings (Hardie et al., 2022). Originally conceptualized by Edmondson as a “shared belief that the team is safe for interpersonal risk taking,” psychological safety enables individuals to ask for help, admit mistakes, or offer ideas without fear of embarrassment or punishment (Edmondson, 1999; Mogård et al., 2022). In higher education, this construct has been increasingly applied to classroom discussion, simulation, and small-group learning, where social dynamics are salient and public performance is often tied to assessment (Frykedal and Chiriac, 2011). When psychological safety is low, students typically engage in self-protection strategies, staying silent, offering only “safe” answers, or avoiding visibility, to minimize the risk of social judgment by peers or instructors (Thomsen et al., 2025). These dynamics become particularly pronounced in activities that combine performance and peer evaluation, because students must manage both academic expectations and their social image simultaneously (Brouwer et al., 2022).

Peer feedback is widely promoted as a student-centered strategy that develops evaluative judgment, critical thinking, and collaboration skills (Zeng and Ravindran, 2025). Compared with instructor-only feedback, peer feedback can be more immediate, dialogic, and pitched at an accessible level. Students often “hear” peers more clearly because they share similar language and learning struggles (Hardman, 2016). Moreover, peer assessment practices align with contemporary competency-based and outcomes-based curricula that expect learners to appraise the work of others and to justify quality criteria (Gielen et al., 2011). However, peer feedback is not a neutral practice. It requires students to position themselves in relation to classmates, to comment on publicly visible performance, and sometimes to assign marks or grades that may affect relationships (Ardill, 2025). For many undergraduates, particularly in collectivist or relationship-oriented cultures, giving critical feedback to peers carries relational risk and can threaten group harmony (Leung et al., 2011). Receiving feedback in front of peers may also trigger concerns about “loss of face,” being exposed as incompetent, or being singled out for weakness (Bok et al., 2013). These experiences point to psychological safety as a mediating condition that determines whether peer feedback becomes developmental or threatening.

Oral and classroom presentations add another layer of vulnerability. Unlike written assignments, presentations are public, temporally fixed, and highly observable; everyone can see mistakes, hesitations, language difficulties, or technology problems in real time (Mardiningrum and Ramadhani, 2022). Performance in such settings is intertwined with students’ self-presentation, confidence, and sense of belonging in the class community (Zengilowski et al., 2023). When peer feedback occurs immediately after a presentation, the student is still in a heightened evaluative state, which may heighten sensitivity to tone, wording, and source of feedback (Sun et al., 2023). Research on social evaluative threat shows that public comparison in front of peers can elicit anxiety, shame, and defensive attributions, which may reduce openness to feedback and willingness to try again (Tsai et al., 2014). Therefore, even well-designed peer-feedback rubrics may not function as intended if the relational climate does not feel safe.

Prior research has largely focused on the psychometric properties of peer assessment, particularly reliability and alignment with instructor grading (Salehi and Masoule, 2017). In contrast, relatively few studies have examined the subjective experience of being evaluated by peers. Ireland (2016), for example, highlighted presentation anxiety but did not specifically examine psychological safety as a central construct (Ireland, 2016). Likewise, while several authors have recommended training students to give constructive feedback, very little empirical work has unpacked how students *interpret* feedback events, who is speaking, who is watching, whether the audience is mixed-gender, how social status or language proficiency shapes feedback acceptance, and how the publicness of the classroom affects perceived safety (Winstone et al., 2017). This represents a meaningful gap: psychological safety is often assumed as a background condition in peer-learning designs, but rarely examined as the *central* phenomenon (Ito et al., 2022).

Moreover, psychological safety is not only an individual perception; it is co-constructed through classroom norms, teacher behavior’s, and peer relationships. Instructors who model respectful critique, normalize mistakes as part of learning, and protect students from ridicule create a protective frame that allows learners to take risks during presentations (Johnson et al., 2020). Conversely, if instructors invite “open feedback” without setting boundaries, students may worry that comments will become personal, comparative, or sarcastic, especially in cohorts where competitiveness or prior conflicts exist (Rosell-Aguilar et al., 2018). Power dynamics among students, senior vs. junior, high achievers *vs.* struggling learners, fluent vs. less fluent speakers, can further modulate whether feedback is received as supportive coaching or as public exposure (Savellon et al., 2024). Understanding these micro-dynamics is essential for designing peer-feedback practices that are both pedagogically rigorous and psychologically safe.

Given universities’ growing reliance on peer assessment to scale feedback, develop graduate attributes, and prepare students for feedback-rich workplaces, it is timely to examine peer feedback not merely as an evaluation technique but as a relational event that can either sustain or erode psychological safety (Carless and Boud, 2018; Tai and Adachi, 2019). By foregrounding students’ own accounts of protection, embarrassment, trust, and audience effects, the present study seeks to illuminate the conditions under which peer feedback on presentations becomes acceptable, actionable, and growth-promoting, and the conditions under which it becomes face-threatening and avoided. Addressing this gap can help educators redesign feedback sessions to uphold psychological safety while preserving the benefits of peer-to-peer learning.

### 1.1 Aim of the study

To explore how university students perceive and negotiate psychological safety when giving and receiving peer feedback on oral/classroom presentations, and to identify the interpersonal, contextual, and instructional factors that make such feedback feel supportive versus exposing.

### 1.2 Research questions

1. How do students describe their experiences of psychological safety (or lack of it) during peer feedback on presentations?
2. What relational and contextual conditions (e.g., who is in the audience, familiarity with peers, grading stakes, instructor role) increase or decrease students’ sense of safety during peer feedback?
3. How does perceived psychological safety influence students’ willingness to disclose weaknesses, accept critical comments, and use peer feedback for improvement?

## 2 Method

### 2.1 Design

This study adopted an interpretive (hermeneutic) phenomenological approach informed by Heideggerian philosophy, which views experience as inseparable from its sociocultural and relational context. Rather than seeking to bracket meaning, interpretive phenomenology aims to understand how individuals make sense of lived experiences through interpretation. This orientation was appropriate because psychological safety during peer feedback is shaped by social relationships, cultural expectations, and power dynamics within the learning environment. The study therefore focused on participants’ meaning-making processes rather than describing experiences as purely pre-reflective phenomena (Neubauer et al., 2019). Peer feedback in presentation-based tasks involves simultaneous performance, social comparison, and informal evaluation, making students’ meaning-making processes central to understanding why some feedback is experienced as developmental while other feedback is experienced as shaming. A phenomenological approach is therefore appropriate to illuminate students’ first-person accounts of threat, trust, audience effects, and protection, and to surface how cultural norms around face, respect, and gendered interaction in Saudi higher education shape feedback encounters. Consistent with the aim of the study—to understand how students negotiate psychological safety during peer feedback—the design prioritized rich, thick narrative accounts over measurement. The research process followed the Standards for Reporting Qualitative Research (SRQR) to ensure clarity of sampling, data generation, analysis, and researcher reflexivity (O’Brien et al., 2014). Consistent with interpretive phenomenology, the analysis emphasized shared meanings and patterns across accounts rather than essential structures of experience.

### 2.2 Study setting and recruitment

The study was conducted in the polyclinics and teaching clinics affiliated with King Faisal University (KFU), Al-Ahsa, Saudi Arabia. These polyclinics serve as training sites for health and health-related students (nursing, applied medical sciences, and selected medical preparatory courses) who are required to prepare and deliver case-based or topic-based presentations that are subsequently discussed with peers. The setting is characterized by a culturally conservative context, gender-segregated or gender-sensitive learning spaces, and a high value placed on respect, modesty, and maintaining peer relationships. In such environments, public critique may be interpreted not only academically but also socially, which makes psychological safety particularly salient. Students were recruited using purposive sampling in collaboration with course coordinators and clinical instructors: an invitation was circulated to students who had recently completed at least one presentation followed by structured or semi-structured peer feedback in the polyclinic. Maximum variation was sought with respect to program (nursing vs. other health tracks), gender, academic level, and presentation type (individual *vs.* group) to capture a broad range of experiences and social dynamics. This diversity enhances the transferability of findings to similar Saudi university clinical teaching settings.

All interviews were conducted in private counselling or meeting rooms within the polyclinics to ensure confidentiality and reduce social pressure. For students on external rotations, interviews were conducted via secure Microsoft Teams in a private setting chosen by the participant.

A polyclinic in the Saudi university context is an outpatient academic training facility where students deliver supervised clinical case discussions and presentations as part of coursework assessment.

### 2.3 Inclusion and exclusion criteria

#### 2.3.1 Inclusion criteria

- Undergraduate student (level 4–8) enrolled in a health or health-related program at King Faisal University.
- Had delivered at least one oral/classroom or case-based presentation in the KFU polyclinics or associated clinical teaching units during the current or immediately preceding semester.
- Had received or provided peer feedback (oral, written, or rubric-based) in the presence of classmates and/or an instructor.
- Able to participate in a 45–90 min interview in Arabic or English.
- Willing to provide informed consent and discuss personal feelings of safety, embarrassment, and peer relations.

#### 2.3.2 Exclusion criteria

- Internship-year students or graduates no longer attending KFU activities.
- Students with documented cognitive or communication difficulties that would impede in-depth interviewing.
- Students who did not experience peer feedback (i.e., instructor-only feedback) on their presentations.
- Students are unwilling to be audio-recorded.

### 2.4 Data collection

Prior to each interview, participants first completed a brief demographic form (program, academic level, gender, and presentation format). The interview then followed using the semi-structured guide. Data were collected through in-depth, semi-structured individual interviews to elicit detailed accounts of how students experienced peer feedback as either supportive or exposing. Semi-structured interviewing was selected because it allows the researcher to ask about potentially sensitive experiences, such as feeling shamed in front of peers or avoiding speaking in mixed groups, while still giving participants space to narrate events in their own words (Bearman, 2019). For students on off-campus rotations, interviews were conducted via secure MS Teams with cameras off if preferred. All interviews were conducted by the first author, a nursing/health-education researcher not involved in students’ assessment, to reduce power imbalances and social desirability. Brief demographic data (program, level, gender, presentation type) were collected before the interview.

### 2.5 Interview structure and conduct

The interview guide was developed from the literature on psychological safety, peer assessment, and classroom public performance and was reviewed by two experts in nursing education and qualitative inquiry. It was piloted with two students from the same university (not included in the analysis) to check clarity and cultural acceptability. The guide used open-ended prompts such as:

- “Tell me about the last time you presented in the polyclinic and your classmates commented on it.”
- “At which moment did you feel most exposed or uncomfortable?”
- “What makes feedback in front of peers feel safe or unsafe for you?”
- “How do the instructor and the class rules affect how you speak to each other?”
- “Would you give the same feedback if the student was from another section/gender/achievement level? Why or why not?”

Probes were used to unpack audience composition, tone of feedback, perceived fairness, and the role of grades. Interviews lasted between 45 and 88 min (average ≈ 62 min). Immediately after each interview, the researcher wrote field notes about non-verbal cues, hesitations around gender or status issues, and contextual features of the classroom or clinic session.

### 2.6 Recording and transcription

With participants’ written and verbal consent, all interviews were digitally audio-recorded using encrypted university devices. Audio files were transferred to a password-protected drive on the KFU server accessible only to the research team. Interviews conducted in Arabic were transcribed verbatim in Arabic and then translated into English by a bilingual research assistant familiar with health-education terminology. A second bilingual team member checked the Arabic–English pairs for fidelity, especially for culturally loaded expressions like “don’t embarrass me,” “in front of the girls,” or “make me small,” to preserve the psychological meaning of exposure. English interviews were transcribed verbatim by a professional transcriber and cross-checked against the recordings by the first author. All transcripts were anonymized using participant codes (e.g., S01, S14) and any potentially identifying references to instructors, courses, or specific polyclinic units were masked.

### 2.7 Data analysis

**Figure 1:**
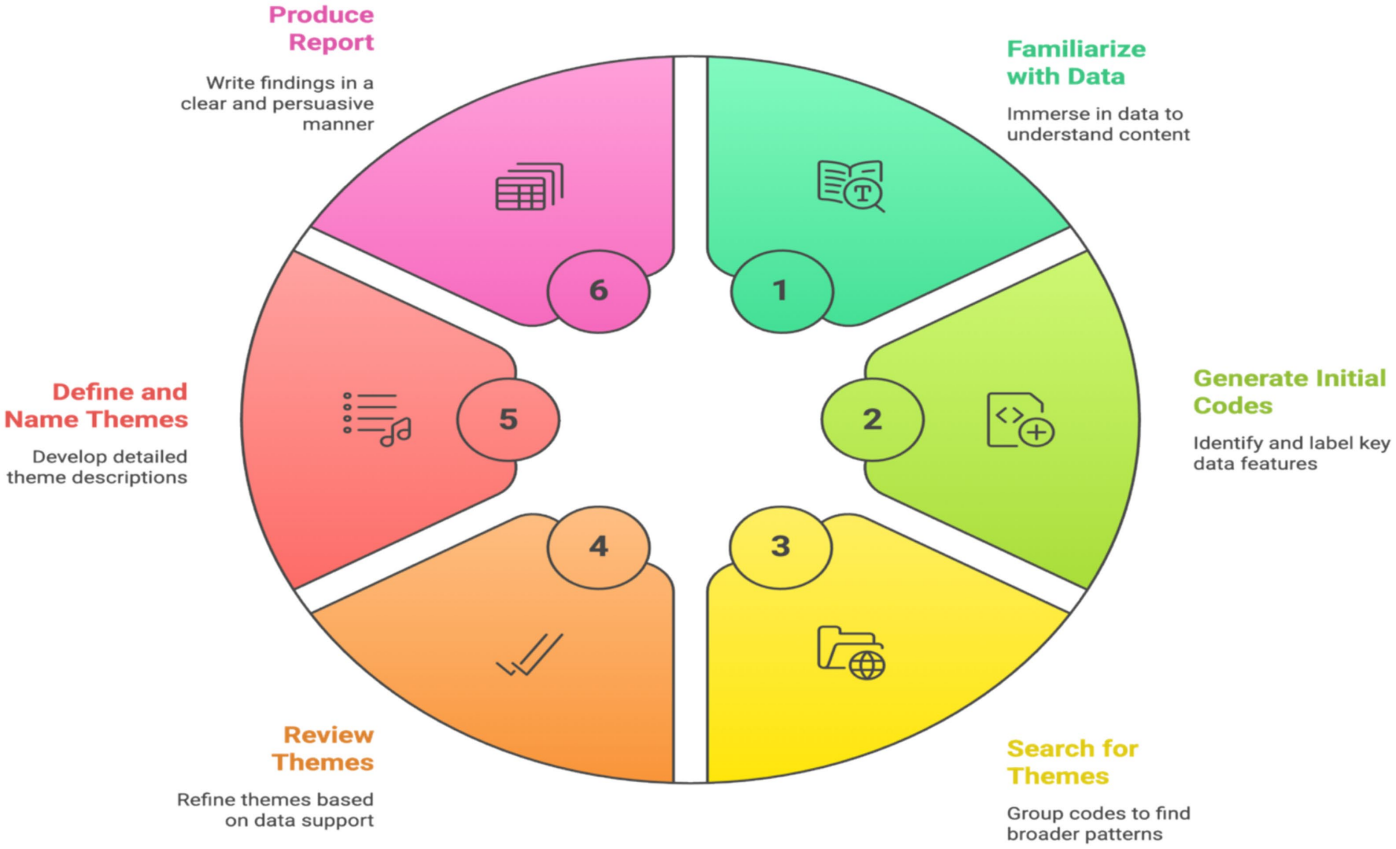
Braun and Clarke’s (2006) six-phase framework.

Data were analyzed thematically using Braun and Clarke’s six-phase framework (Braun and Clarke, 2022, 2025), supported by NVivo 14 (Figure 1). First, all transcripts and field notes were read repeatedly to gain familiarity with the accounts and to note first impressions about “publicness,” “saving face,” and “who is watching.” Second, initial codes were generated inductively at the semantic and latent levels (e.g., “protecting peers,” “fear of negative comparison,” “teacher as shield,” “gender boundaries”). Third, codes were collated into candidate themes such as “feedback as relational work,” “conditions for safe critique,” and “performing competence under observation.” Fourth, themes were reviewed against the entire dataset to check coherence and distinctiveness. Fifth, themes were defined and named, with attention to how they explained the core phenomenon of psychological safety in peer feedback. Finally, a narrative analytic account was produced, using compelling extracts to illustrate each theme. Reflexive memos were integrated throughout to track analytic decisions.

### 2.8 Ethical considerations

Ethical approval was obtained from the King Faisal University Research Ethics Committee. All students received an information sheet outlining the purpose of the study, the voluntary nature of participation, and the right to withdraw without academic consequences. Because the topic involves discussing classroom dynamics and comments made by identifiable peers, participants were reminded not to disclose classmates’ real names during the interview; where names were mentioned, they were removed at transcription. Written informed consent was obtained before data collection. Audio files, transcripts, and consent forms were stored separately on encrypted drives in accordance with KFU data-protection policies. Findings are reported in aggregate to reduce deductive disclosure, particularly for small, gender-segregated cohorts.

### 2.9 Rigour and reflexivity

Rigor was addressed using reflexive thematic analysis quality principles rather than validation-based criteria. Consistent with Braun and Clarke (2022), emphasis was placed on researcher reflexivity, transparency of analytic decisions, and depth of interpretation rather than consensus or accuracy checking. Credibility was enhanced through member checking: six participants were invited to review a summary of emerging themes about “exposure,” “supportive peers,” and “instructor buffering,” and all confirmed that the account reflected their experiences (Guba and Lincoln, 2001). Dependability was supported by maintaining an audit trail including the interview guide versions, reflexive memos, coding frameworks, and NVivo export files. Confirmability was promoted through peer debriefing with two qualitative researchers not involved in data collection, who examined coding–theme links and challenged culturally biased interpretations. Transferability was facilitated by providing a thick description of the KFU polyclinic teaching context, gendered learning spaces, and assessment expectations so that educators in similar Gulf/Middle Eastern universities can judge applicability. Throughout the study, the first author kept a reflexive journal to monitor positionality as a faculty member in the same institution and to bracket assumptions about “good feedback,” instructor authority, and student deference.

## 3 Results

### 3.1 Characteristics of participants

Eighteen undergraduate students enrolled in health and health-related programs at King Faisal University, Al-Ahsa, participated in the study (Table 1). Participants were aged between 19 and 24 years, with a mean age of 21.3 years (SD = 1.4). The sample included 10 female and 8 male students, reflecting the gender-segregated streams and clinical teaching arrangements in the university polyclinics. Most participants were registered in the nursing program (*n* = 9), followed by applied medical sciences tracks such as respiratory therapy and medical laboratory sciences (*n* = 5), and other health-related programs (clinical nutrition and health education; *n* = 4). The majority were in mid-to-senior academic levels (levels 5–7), when presentation-based clinical discussions and peer critique are commonly used to assess professional communication, case analysis, and teamwork skills.

**Table 1:** Demographic and academic characteristics of participants (*n* = 18).

Participant ID	Age range (yrs)	Gender	Program	Academic level	Presentation type	Peer feedback format	Audience/session type
S01	19–20	Female	Nursing	Level 5	Individual	Oral + rubric	Female section
S02	21–22	Male	Nursing	Level 6	Group	Oral	Male section
S03	20–21	Female	Clinical Nutrition	Level 5	Individual	Oral + written (collected)	Female section
S04	22–23	Male	Respiratory Therapy	Level 7	Individual	Oral + rubric	Mixed/inter professional
S05	19–20	Female	Nursing	Level 4	Group	Oral	Female section
S06	21–22	Female	Nursing	Level 6	Individual	Oral + rubric	Female section
S07	23–24	Male	Medical Laboratory	Level 7	Individual	Oral	Mixed/inter professional
S08	20–21	Female	Nursing	Level 6	Group	Oral + rubric	Female section
S09	21–22	Male	Applied Med Sci	Level 5	Individual	Oral	Male section
S10	19–20	Female	Health Education	Level 5	Individual	Oral + rubric	Female section
S11	22–23	Male	Nursing	Level 7	Group	Oral	Male section
S12	21–22	Female	Nursing	Level 6	Individual	Oral + written (collected)	Female section
S13	20–21	Male	Respiratory Therapy	Level 5	Individual	Oral + rubric	Mixed/inter professional
S14	21–22	Female	Nursing	Level 7	Group	Oral	Female section
S15	22–23	Male	Medical Laboratory	Level 6	Individual	Oral + rubric	Male section
S16	20–21	Female	Clinical Nutrition	Level 5	Individual	Oral	Female section
S17	23–24	Male	Nursing	Level 7	Group	Oral	Mixed/inter professional
S18	21–22	Female	Nursing	Level 6	Individual	Oral + rubric	Female section

All students had delivered at least one oral or case-based presentation in the KFU polyclinics during the current or immediately preceding semester, and all had taken part in peer feedback in front of classmates and an instructor. 11 participants reported presenting individually, while seven had presented as part of a small group (3–5 students). Peer feedback was delivered in several formats: all 18 experienced live, oral comments from peers in a plenary setting; 12 received rubric-based peer scoring; and 4 had also experienced partially anonymous written comments collected by the instructor. 12 participants reported that their feedback sessions took place in single-gender groups (female-only or male-only), whereas six described mixed or inter professional sessions in which students from different tracks or genders were present—these students more frequently described heightened feelings of possible “exposure.” Recruitment continued until sufficient information power was achieved, meaning additional interviews did not substantially alter the analytic interpretation (Braun and Clarke, 2022).

### 3.2 Thematic findings

Thematic analysis generated four interconnected themes that illuminate how students experienced psychological safety (or its absence) during peer feedback on presentations in the polyclinics of King Faisal University. Together, these themes show that students did not reject peer feedback as a learning strategy; rather, they evaluated *where*, *by whom*, and *in front of whom* feedback was delivered. Psychological safety was constantly negotiated against the risks of public exposure, loss of face, gendered expectations, and future peer relations. Instructor behaviors, audience composition, and feedback format emerged as decisive contextual levers that could either turn feedback into a constructive learning moment or a socially threatening event Table 2.

**Table 2:** Themes, sub-themes, and descriptions.

Theme	Sub-theme	Description
1. “Please do not expose me”: public feedback as social threat	1.1 Publicness amplifies vulnerability	Students perceived feedback given immediately after a presentation, in front of the whole section, as the riskiest moment; mistakes were still “fresh,” and everyone could see who was being corrected. Exposure was felt more strongly in mixed/inter professional sessions.
	1.2 Protecting face and peer image	Learners were highly concerned about being seen as weak, unprepared, or linguistically inadequate. Fear of ridicule or future gossip made some students withhold questions, speak less, or give very soft feedback to others to avoid reciprocal exposure.
2. Instructor as safety broker	2.1 Framing and moderating feedback	When instructors set clear rules (“focus on the work, not the person,” “one strength, one suggestion”), students reported feeling safer to listen and to speak. Structured turn-taking reduced the risk of harsh or personal comments.
	2.2 Shielding from harsh peers	Students relied on the instructor to intervene when feedback became evaluative or comparative (“your slides were worse than X”), especially in male groups. Where teachers did not mediate, students interpreted the space as unsafe and became defensive.
3. Relational calculus in giving feedback	3.1 Feedback filtered through relationships	Students evaluated whether the peer was a close friend, high-achieving student, or from another section. Critical feedback was softened for friends and for peers who seemed anxious; stricter comments were reserved for confident or high-status students.
	3.2 Gender and cultural sensitivities	In gender-sensitive or mixed sessions, students avoided direct critique to prevent embarrassment across gender lines. Female students, in particular, preferred private or written comments to avoid being associated with “shaming” a colleague.
4. Making peer feedback safe and useful	4.1 Quiet/anonymous channels enable honesty	Written, collected, or partially anonymous peer comments were described as “easier to accept” because they removed the audience and reduced face-threat. Students said they could be more specific and action-focused in these formats.
	4.2 Growth-oriented framing encourages engagement	When feedback was clearly linked to clinical communication skills, future rotations, or exam performance, students tolerated critique better and viewed it as professional—not personal—evaluation. Praising first, then suggesting change, was repeatedly named as the safest pattern.

#### 3.2.1 “Please don’t expose me”: public feedback as social threat

This theme captures students’ descriptions of peer feedback as a socially high-stakes event—less about the technical accuracy of comments and more about *where* and *in front of whom* they were delivered. Participants consistently portrayed the moments immediately following a presentation as emotionally charged: attention was still fixed on the presenter, adrenaline was high, and any critique—however well-intended—felt amplified. Students weighed the pedagogical value of critique against potential social costs: damaged reputation, future teasing, or being remembered for a single flaw rather than overall competence. The result was a pervasive vigilance—scanning the room, anticipating who might speak, and bracing for comments. When sessions were tightly framed and moderated by instructors, learners were more willing to listen and respond; when they were open-ended, feedback could slide toward comparison, sarcasm, or personal tone, which students experienced as exposure rather than support.

##### 3.2.1.1 Publicness amplifies vulnerability

Students repeatedly linked the *publicness* of feedback to a felt loss of control over their academic and social image. Being corrected “on the spot,” with classmates watching, was described as the point of maximum vulnerability. Several participants emphasized the difference between hearing the same suggestion privately versus publicly—public delivery changed the meaning of the message from “help” to “judgment.” Mixed or inter professional audiences intensified this effect because relationships were newer and social norms less predictable.

One participant described the immediacy of public attention as intensifying the emotional impact of feedback: “When the whole room is still looking at me and someone points out a mistake, it doesn’t feel like advice—it feels like a label.” (S04)

Another added “If the comment is written, I can breathe and think. When it’s said out loud, I only hear the “in front of everyone” part.”

(S12) also another added “In mixed sessions I’m extra careful. I don’t know how they will talk about it later outside the class.” (S07).

Several students described “performing safety”—smiling, nodding, or agreeing quickly to end the public moment—then privately discounting the feedback. Others pre-empted risk by reading more from slides, speaking faster, or simplifying content to avoid mistakes that might be highlighted. A few participants reported strategically volunteering positive comments for peers so that the tone of the session stayed supportive, hoping the same courtesy would be returned. Publicness thus shaped not only how feedback was *received* but also how presentations were *delivered*, often narrowing opportunities for authentic performance and learning.

##### 3.2.1.2 Protecting face and peer image

Concern for “face” and ongoing peer relationships guided how students both *gave* and *edited* feedback. Learners described a constant calculation: the peer’s status (strong/struggling), closeness (friend/acquaintance), likely sensitivity, and the social memory of the cohort. Many preferred indirect language (“maybe consider,” “one thing to improve”) or generic praise before a single suggestion to avoid appearing harsh. Female students, in particular, highlighted the risk of being seen as the person who “embarrasses others,” and favored written or instructor-curated channels when critique had to be specific.

One participant described “I don’t want to make her small. I start with what went well and keep one suggestion only.” (S01)

Another aside “If he’s my friend, I know how to say it. If not, I keep it very light because I don’t want problems later.” (S11)

“Sometimes I write what I really think and give it to the instructor to pass, so it helps without making her feel exposed.” (S03).

This face-protective logic also influenced *acceptance* of feedback. Comments from respected or “safe” peers were easier to absorb; remarks from competitive classmates were more likely to be read as performative or comparative, even when content was valid. Students described valuing feedback that targeted the *work* (structure, evidence, slide design) rather than the *person* (ability, confidence). When instructors modeled and enforced that boundary—e.g., asking for “one strength, one actionable suggestion”—students reported greater willingness to disclose weaknesses and request help. In short, protecting face was not mere avoidance; it was an adaptive strategy to keep relationships intact while still extracting learning—best enabled when formats and facilitation lowered the social cost of being corrected.

#### 3.2.2 Instructor as safety broker

This theme highlights the pivotal role of the instructor in either activating or neutralizing psychological safety during peer feedback. While peer feedback was the formal pedagogy, students repeatedly described the *instructor*—not classmates—as the person who determined “how safe” the session would feel. When teachers framed feedback as a collective improvement exercise, modeled respectful language, and controlled turn-taking, students reported being more open to hearing critique and even more willing to offer it. Conversely, when instructors took a hands-off approach (“any comments?”) without boundaries, students perceived the space as unpredictable, leaving room for competitive, overly personal, or performative peer comments. In such cases, learners shifted into self-protection and either minimized their participation or discounted the feedback afterward. Thus, the instructor functioned as a *safety broker*—actively constructing the conditions in which peer-to-peer critique could occur without threatening students’ social standing.

##### 3.2.2.1 Framing and moderating feedback

Students emphasized that what the instructor said *before* the feedback started often mattered more than the feedback itself. Clear framing—“we are commenting on the presentation, not the presenter,” “we start with positives,” “every comment must include a suggestion”—signaled to students that the session had rules, that harshness would not be rewarded, and that everyone would be treated equally. This advance structuring reduced anxiety, especially for students who had previously experienced unmoderated critique.

One participant described, “When the doctor explains the goal first, I relax. I know nobody will attack me because he already said how to talk.” (S06). Another added, “She made us follow the rubric. That helped. We focused on the slides and objectives, not on the person.” (S10).

Moderation during the session was equally important. Instructors who summarized, rephrased, or “translated” student comments into constructive language modeled how to give feedback without causing loss of face. For example, a blunt peer remark (“your English was not clear”) was reformulated by the instructor into a skill-focused suggestion (“next time, slow your pace so the good content is clearer”). Students reported that this kind of real-time linguistic scaffolding turned a potentially embarrassing moment into an actionable one. It also taught them the discourse of professional feedback, which they could then imitate in later sessions. In short, framing and moderating were not cosmetic; they were the mechanisms through which the instructor converted a socially risky practice into a pedagogically safe one.

##### 3.2.2.2 Shielding from harsh peers

Alongside framing, students expected the instructor to *protect* them when peer comments crossed an implicit boundary—became too personal, comparative, or delivered with the wrong tone. Several participants narrated incidents where a classmate used feedback time to display superiority or to reopen a prior tension; in these moments, the instructor’s intervention (or lack of it) was read as a signal of how much the student was “worth protecting.”

One participant described “Sometimes one student talks like he is the teacher. If the doctor doesn’t stop him, we feel unsafe.” (S15).

One participant described “He corrected me in a way that made the class laugh. The instructor said, ‘focus on the content, please,’ and that saved me.” (S04).

Where instructors stepped in—by stopping the comment, softening its wording, or redirecting it to the rubric—students felt seen and respected. This protection encouraged them to participate again in future sessions because they trusted that “someone is watching the line.” By contrast, when instructors remained silent during a harsh or mocking comment, students concluded that the classroom was not a reliably safe space. Some said they would “prepare to defend,” others said they would avoid volunteering presentations, and a few admitted they would give only “nice” feedback to stay off others’ radar. In other words, shielding was not merely kindness; it preserved the *function* of peer feedback as a learning tool. Without the instructor as a shield, students reoriented their behavior around social survival rather than academic growth, weakening the very purpose of peer assessment.

#### 3.2.3 Relational calculus in giving feedback

This theme describes how students weighed interpersonal histories, status hierarchies, and future consequences before deciding *what* to say and *how* to say it during peer feedback. Rather than a neutral exchange of observations, feedback operated as a relational negotiation: learners asked themselves whether the recipient was a close friend, a high achiever, an anxious presenter, or someone with whom they might collaborate again. These calculations shaped the specificity, directness, and even the *channel* of feedback (spoken vs. written). Students aimed to preserve working harmony and avoid retaliatory or reputational fallout, sometimes at the expense of precision. As a result, the same rubric could produce markedly different comment patterns depending on who was involved and how relationships were read in that moment.

##### 3.2.3.1 Feedback filtered through relationships

Participants consistently reported softening or reframing critique for friends or visibly nervous peers, while being more candid with confident classmates perceived as “able to handle it.” Anticipated future interactions also mattered; students avoided harsh comments if they expected to be grouped with the same person in coming rotations. Trust accelerated candor: when a history of supportive exchanges existed, students felt licensed to be more specific and technical.

One participant described “With my friend I can say, “your objectives didn’t match the results.” She knows it’s for improvement. With others I say, “overall good,” because I don’t want issues later.” (S14)

“If someone looked stressed, I chose one small point only. No list—just one thing to fix.” (S05)

One participant described “When I know we’ll work together again, I avoid anything that can create tension. I keep the details for a private message.” (S11).

Relational filtering also influenced *who* spoke. Some students deferred to high-status peers (top scorers, fluent speakers) to set the tone, then aligned their comments accordingly. Conversely, when a dominant peer gave sharp criticism, others stayed quiet to avoid being seen as oppositional. In practice, this produced a feedback ecology where the *network* of relationships, not just the rubric, governed the granularity and balance of comments.

##### 3.2.3.2 Gender and cultural sensitivities

Gender norms and broader cultural expectations about respect and modesty further shaped feedback choices. Many students—especially women—described a strong preference for written or instructor-curated channels when commenting across gender lines to avoid public embarrassment or misinterpretation. Public critique of the opposite gender risked being read as personal rather than academic, with potential social repercussions beyond the classroom.

One participant described “I wouldn’t correct him directly in front of everyone. I write it and give it to the instructor so it helps without making him lose face.” (S03)

Another added “For a male student, I keep it very general. Detailed points I send later in writing.” (S08)

“In mixed sessions, we are extra careful with words. Even a good suggestion can sound like an attack if said the wrong way.” (S07).

Cultural sensitivities also intersected with language proficiency and public speaking confidence. Students avoided comments that might highlight accent, fluency, or demeanor, viewing these as identity-adjacent and thus risky to address publicly. Instead, they focused on task features (slide structure, time management, reference use) that could be framed as professional skill-building. When instructors reinforced this boundary—“critique the work, not the person”—students reported greater ease giving actionable suggestions across gender and social lines. In sum, gendered and cultural considerations were not barriers to feedback but *parameters* within which students managed dignity and belonging; when respected, these parameters enabled honest, skill-focused critique without social harm.

#### 3.2.4 Making peer feedback safe and useful

This theme shows that students did not oppose peer feedback itself; what they resisted was *high-cost* feedback—public, personal, or delivered in an unpredictable tone. When the delivery format lowered social risk, students became noticeably more honest as givers and more receptive as receivers. They repeatedly contrasted “feedback in front of everyone” with “feedback I can read later,” describing the latter as calmer, more actionable, and less tied to saving face. At the same time, students were more willing to tolerate critique when it was clearly positioned as part of their professional formation—“this is how nurses/health students improve”—rather than as a judgment of personal ability. In other words, psychological safety and academic rigor were not opposites; the right structures made rigorous feedback *possible*.

##### 3.2.4.1 Quiet/anonymous channels enable honesty

Students strongly favored feedback channels that removed the audience or disguised the source—written slips collected by the instructor, online forms, rubric checklists, or instructor-synthesized comments. These “quiet” routes allowed peers to be specific (“slide font too small,” “not linked to objectives,” “unclear case rationale”) without fearing a hurt reaction or future tension. They also allowed recipients to process comments privately, away from the gaze of classmates.

One participant described “When it is written, people tell the truth. When it is public, they only say “very good”.” (S10)

Ine student added “The doctor collected our notes, then read them without names. I could accept it because no one was looking at me.” (S02)

Also “Anonymous is better because I don’t want her to think I am against her.” (S16).

Several participants said they actually *learned more* from anonymous peer comments because they received multiple perspectives at once and because the wording was often more direct. Quiet channels also leveled power dynamics—shy students, junior students, or those from other tracks could contribute feedback without having to “take the floor.” Importantly, anonymity was not only about hiding identity; it was about *decoupling* feedback from social relationships so the message could stand on its own.

##### 3.2.4.2 Growth-oriented framing encourages engagement

Students were far more accepting of critique when it was explicitly linked to improvement, professional standards, or upcoming assessments. When instructors framed peer feedback as “training for clinical presentations,” “preparation for OSCEs,” or “what supervisors will expect,” students interpreted even corrective comments as legitimate and future-oriented, not as personal attacks.

“When she said, “this is how you will present for the exam,” I listened even if it was negative.” (S08)

“If the comment shows me what to do next time, I don’t feel exposed—I feel guided.” (S13).

Growth-oriented framing also influenced *how* students gave feedback. Many described using a “positive first, suggestion next” pattern because it preserved dignity while still moving the peer forward. Others said they tried to tie comments to rubrics (“you met this, you need this”) so they would be seen as objective rather than opinion. Where instructors summarized feedback into two or three action points—“slow your pace,” “align objectives,” “add references”—students said the session felt organized, fair, and safe. In such environments, psychological safety did not mean avoiding critique; it meant structuring critique so that it pointed to the next, achievable step.

## 4 Discussion

The findings of this study show that peer feedback on oral presentations is not experienced by students as a simple pedagogical exercise but as a socially negotiated event in which psychological safety must first be secured before learning can occur. At the core of the phenomenon was students’ plea, explicit in several interviews, of “please don’t expose me,” which mirrors Edmondson’s description of psychological safety as freedom from the fear of embarrassment or punishment in front of a salient group (Prosenjak and Lučev, 2020). In our context, exposure was not only academic (being corrected) but also relational and reputational (being corrected *publicly*). This helps explain why, despite the well-established benefits of peer assessment for developing evaluative judgment and self-regulation (Tai, 2018), students sometimes respond with defensiveness, surface participation, or overly positive feedback: they are protecting social capital in a cohort that they must keep studying with (Brouwer et al., 2016).

The first theme, public feedback as a social threat, confirms that the *context of delivery* can undermine even well-designed feedback tools. Students said openly that the same comment was acceptable on paper but humiliating when voiced immediately after the presentation. This aligns with work on social evaluative threat, showing that attention from an observing audience amplifies shame and decreases receptivity to critique (Hofmann, 2000). It also resonates with the argument that trust is the missing component in many peer feedback designs: without trust, students interpret feedback through the lens of status, competition, or social comparison rather than improvement (Ait Taleb, 2024). In Saudi and other collectivist, relationship-oriented settings, where “face” and cohort harmony carry high value, publicness magnifies this interpretive lens. Thus, the issue for these students was not that peers could not judge quality, but that open, synchronous, audience-facing critique carried too high a social price.

Equally striking was the second theme, *instructor as safety broker*. Although peer feedback is often promoted to reduce teacher dominance, our participants still relied on the instructor to set the emotional temperature of the session. When teachers framed the purpose (“we help each other for future clinical presentations”), enforced tone (“focus on the work, not the person”), and rephrased clumsy student comments, peers felt licensed to be honest, and recipients stayed engaged. This is consistent with Boud and Molloy’s notion of “feedback designs,” where the teacher’s main role is to create relational and procedural conditions in which feedback can circulate productively (Molloy et al., 2020). Our data suggest that, in this context, *withdrawing* too much teacher presence in the name of peer learning may actually reduce the quality of peer feedback, because students interpret unmoderated space as risky (Lerchenfeldt et al., 2019). The instructor, therefore, is not made redundant by peer assessment; s/he is repositioned as guarantor of dignity (Jiang et al., 2023).

The third theme, *relational calculus in giving feedback,* adds an underexplored layer to the peer-assessment literature. Much research has focused on reliability and alignment with teacher marks (van Zundert et al., 2010), but fewer studies have examined how friendship, anticipated future collaboration, and internal cohort politics *filter* feedback before it is even spoken. Our participants admitted to intentionally diluting or redirecting critique to preserve relationships, particularly when the presenter seemed anxious or when the cohort was small and stable. This is an intelligent, prosocial move from the students’ perspective, but it has methodological consequences: it means that feedback quality is not only a function of students’ assessment literacy, but also of their social risk analysis (Hübner and Pfost, 2024; Poulos and Mahony, 2008). Cultural and gendered considerations intensified this: female students, and those commenting across gender lines, preferred private or anonymized routes to avoid being seen as the person who “causes embarrassment,” a finding that echoes work on feedback and face-management in Asian and Middle Eastern classrooms (My, 2021). Program me leaders in such settings should therefore avoid assuming that oral, plenary peer critique will automatically produce rich, specific comments.

The final theme, *making peer feedback safe and useful,* is important because it shows a route *forward* rather than merely describing barriers. Students were very clear about what worked: collecting written feedback and having the instructor read or synthesize it; using rubrics to depersonalize critique; sequencing praise before suggestions; and, above all, framing the feedback as preparation for real clinical, OSCE, or workplace presentations. When comments were visibly tied to future professional performance, students reclassified them from “personal attack” to “legitimate standard,” which is consistent with Carless and Bound’s argument that learners need feedback literacy, understanding the purpose, processes, and use of feedback, to act on it (Carless and Boud, 2018). Quiet or semi-anonymous channels also democratized participation: weaker, shy, or non-dominant students could finally say what they saw, which improves the validity of peer feedback overall (Ting, 2024). In other words, psychological safety was not about removing critique; it was about sequencing and packaging critique so that it could be heard (Silva et al., 2025).

### 4.1 Limitations of the study

This study was conducted in a single Saudi university context (King Faisal University polyclinics), where learning is shaped by gender-sensitive arrangements and strong relational norms; transferability to coeducational or more anonymous higher-education settings should therefore be made cautiously. Data relied on self-reported interviews; students may have downplayed episodes of ridicule or conflict to protect peers or faculty, introducing social desirability bias. Because only students who had actually presented and received peer feedback were included, the perspectives of those who avoid presenting—arguably the least psychologically safe group—were not captured. Finally, the study did not triangulate interview data with classroom observations, so moment-to-moment instructor mediation of feedback could not be directly verified.

Students reflecting on experiences from the previous semester may have differed in recall accuracy compared with those describing recent presentations.

### 4.2 Implications for nursing education and practice

The findings indicate that peer feedback on presentations should be intentionally structured to minimize face-threat. Nursing educators can adopt a two-stage model: brief, moderated public comments (strength + 1 suggestion) followed by anonymous or written peer feedback that is synthesized by the instructor. Clear framing—“we critique the work, not the person”—and instructor rephrasing of clumsy comments should be treated as core teaching skills, not optional courtesies. Programs should also embed feedback literacy in undergraduate nursing curricula, teaching students how to give behavior-focused, rubric-based, and culturally sensitive feedback that preserves dignity while advancing clinical communication competence. In clinical teaching and inter professional sessions, where mixed groups heighten exposure, tighter facilitation and use of written channels can help ensure participation from quieter or less powerful students. Ultimately, creating psychologically safe feedback environments will help nursing students rehearse the kind of constructive, non-shaming feedback expected in real clinical teams.

## 5 Conclusion

Peer feedback on oral presentations can be a powerful learning strategy for nursing and health students, but only when psychological safety is deliberately established. Students in this study evaluated feedback not by its technical correctness but by its social cost—who said it, in front of whom, and how it was mediated. Instructors played a decisive “safety broker” role, and formats that removed public exposure made feedback more honest and more usable. Designing feedback practices that respect local cultural and gender norms, while still maintaining academic rigor, is therefore essential to producing reflective, communicative, and team-ready nursing graduates.

## Data Availability

The datasets presented in this article are not readily available because they contain potentially identifiable student responses on a sensitive topic, and public sharing could compromise participant anonymity and confidentiality under the study’s ethics requirements. Requests to access the datasets should be directed to seali@kfu.edu.sa.
