# Correction: “Please don't expose me”: students' perceptions of psychological safety during peer feedback on presentations

**DOI:** 10.3389/fpsyg.2026.1870148

**Published:** 2026-06-08

**Authors:** Suzanne Mohamed Arafa, Sayed Ibrahim Ali

**Affiliations:** 1 Mohammed Jaber Al Ansari College for Teachers, University of Bahrain, Manama, Bahrain; 2 Educational Psychology Department, Faculty of Education, Capital University (Helwan), Cairo, Egypt

**Keywords:** oral presentations, peer feedback, psychological safety, qualitative phenomenology, Saudi Arabia, student perceptions

Affiliation “Mohammed Jaber Al Ansari College for Teachers, University of Bahrain, Manama, Bahrain” was omitted from the paper. This affiliation has now been added for author Suzanne Mohamed Arafa.

Author Suzanne Mohamed Arafa was erroneously omitted as corresponding author. The correct corresponding authors are Sayed Ibrahim Ali and Suzanne Mohamed Arafa.

The original version of this article has been updated.

